# A New Insight into Flowering Regulation: Molecular Basis of Flowering Initiation in *Magnolia* × *soulangeana* ‘Changchun’

**DOI:** 10.3390/genes11010015

**Published:** 2019-12-23

**Authors:** Zheng Jiang, Liyong Sun, Qiang Wei, Ye Ju, Xuan Zou, Xiaoxia Wan, Xu Liu, Zengfang Yin

**Affiliations:** 1 Co-Innovation Center for Sustainable Forestry in Southern China, College of Biology and the Environment, Nanjing Forestry University, Nanjing 210037, China; jz19901202@126.com (Z.J.); sunly@njfu.edu.cn (L.S.); jy03231995@163.com (Y.J.); zouxuan1996@126.com (X.Z.); wanxx1996@126.com (X.W.); yiery4125@163.com (X.L.); 2 Bamboo Research Institute, Nanjing Forestry University, Nanjing 210037, China; weiqiang@njfu.edu.cn

**Keywords:** *Magnolia* × *soulangeana* ‘Changchun’, spring and summer flowering initiation, BGISEQ-500 sequencing platform, gibberellin signaling, transcription factor

## Abstract

*Magnolia × soulangeana* ‘Changchun’ are trees that bloom in spring and summer respectively after flower bud differentiation. Here, we use phenological and morphological observation and RNA-seq technology to study the molecular basis of flowering initiation in ‘Changchun’. During the process of flowering initiation in spring and summer, the growth of expanded flower buds increased significantly, and their shape was obviously enlarged, which indicated that flowering was initiated. A total of 168,120 expressed genes were identified in spring and summer dormant and expanded flower buds, of which 11,687 genes showed significantly differential expression between spring and summer dormant and expanded flower buds. These differentially expressed genes (DEGs) were mainly involved in plant hormone signal transduction, metabolic processes, cellular components, binding, and catalytic activity. Analysis of differential gene expression patterns revealed that gibberellin signaling, and some transcription factors were closely involved in the regulation of spring and summer flowering initiation in ‘Changchun’. A qRT-PCR (quantitative Real Time Polymerase Chain Reaction) analysis showed that BGISEQ-500 sequencing platform could truly reflect gene expression patterns. It also verified that *GID1B *(*GIBBERELLIN INSENSITIVE DWARF1 B*), *GID1C*, *SPL8* (*SQUAMOSA PROMOTER BINDING PROTEIN-LIKE 8*)*,* and *GASA* (*GIBBERELLIC ACID-STIMULATED ARABIDOPSIS*) family genes were expressed at high levels, while the expression of *SPY* (*SPINDLY*) was low during spring and summer flowering initiation. Meanwhile, the up- and down-regulated expression of, respectively, *AGL6* (*AGAMOUS-LIKE 6*) and *DREB3* (*DEHYDRATION-RESPONSIVE ELEMENT-BINDING PROTEIN 3*), *AG15*, and *CDF1* (*CYCLIC DOF FACTOR 1*) might also be involved in the specific regulation of spring and summer flowering initiation. Obviously, flowering initiation is an important stage of the flowering process in woody plants, involving the specific regulation of relevant genes and transcription factors. This study provides a new perspective for the regulation of the flowering process in perennial woody plants.

## 1. Introduction

Flowering is a very complicated process in angiosperm plants that is affected by both genetic and environmental factors [1]. Work carried out over recent decades has shown much research progress. Using Solexa/Illumina sequencing platform, researchers have identified almost all transcripts involved in flowering from the vegetative and flower buds in *Dimocarpus longan*, including putative homologues of DELLA protein, *LFY* (*LEAFY*), *SOC1* (*SUPPRESSOR OF OVEREXPRESSION OF CO1*) [2]. Meanwhile, compared to the apical meristem of vegetative growth, gibberellin 3 beta-hydroxylase 2 and LFY were both expressed higher in early flowering, indicated that GA pathway may be related to the early flowering of *Angelica sinensis* [3]. By comparing the differences in transcript expression levels between flowering and non-flowering Moso bamboo samples at different flowering developmental stages, researchers found that GAMYB, *GID1* (*GIBBERELLIN INSENSITIVE DWARF1*), and *GID2* were significantly up-regulated, while DELLA protein was down-regulated, and believed that bamboo flowering was associated with gibberellin signaling pathway [4]. Transcription factors (i.e., SPL (SQUAMOSA PROMOTER BINDING PROTEIN-LIKE) and MYB (myeloblastosis)) and flowering genes (i.e., *FT* (*FLOWERING LOCUS T*), *FD* (*FLOWERING LOCUS D*)) were observed to play key roles in the floral induction in *Malus domestica* [5]. Moreover, Roche/454 sequencing was used to understand the pistillate flowering in *Carya cathayensis*, which found that the flowering event of pistillate flower buds in hickory was triggered by several pathways synchronously, including GA pathway [6]. Using reblooming, non-re-blooming, and wild-type tree peonies (*Paeonia suffruticosa*), Wang et al. pointed that *PsGID1* was important GA signaling genes, and suggested that GA pathway played an important role in the flowering regulation of tree peony [7]. Similar research results were also found in *Populus* L. [8], *Prunus mume* [9], *Poncirus trifoliata* [10] and other woody plants. However, to date, the understanding of flowering regulation in woody plants has been based on the molecular mechanisms underlying the regulation of flower bud differentiation.

In fact, for temperate perennial woody plants with multiple flowering, the buds formed by reproductive transition can flower after a period of dormancy [11,12]. Taking apical buds and flowers from *Malus* × *domestica* ‘Royal Gala’ as materials, researchers found that *MdDAMb* (*DORMANCY ASSOCIATED MADS-BOX*)*, MdSVPa,* and *MdSVPb* played a role in maintaining bud dormancy, and *SVP* (*SHORT VEGETATIVE PHASE*) might play a role in floral meristem identity [13]. The increased level of *PpEBB* (*EARLY BUD-BREAK*) induced high levels of transcription of four *PpCYCD3* (*D-type CYCLIN*) genes, which promoted cell division and resulted in a significant increase in the primordia size of *Pyrus pyrifolia* ‘Kosui’ lateral flower buds [14]. The profile of differentially expressed genes (DEGs) suggests that orthologs of *FT*, *FD*, *TFL1* (*TERMINAL FLOWER 1*), *LFY* (*LEAFY*), and MADS-box genes were the major genes involved in chilling-mediated *Vaccinium corymbosum* bud-break [15]. Obviously, the identification of genes associated with flowering and dormancy release was due to the comparison of reproductive organs in different seasons. It is worth noting that although the flower buds of temperate tree species are differentiated in the first year, and flowering in the next year, a few woody plants can flower many times in one year [16], and the tropical woody plants can even flower after floral transition within a year [17]. The flowering process of trees has two distinctly independent stages: floral transition and flowering initiation [18]. Therefore, it is worthwhile to further study genes related to flowering initiation.

Magnolias are shrubs and trees distributed widely and are relatively ancient. Their ornamental characteristic has been noted because of their large flower and fragrant aroma. In recent years, study of the reproductive organs of Magnolias more commonly focuses on the origin and systematic evolution of flowers [19,20], as well as the physiological and biochemical regulation mechanism, and so on [21,22]. It has been demonstrated that *AP3* (*APETALA 3*) homologues, *AGL* (*AGAMOUS-LIKE*) genes, and other transcription factors play an important role in regulating flower bud differentiation and the development in *Magnolia wufengensis* [23,24] and *Magnolia sinostellata* [25]. However, analyzing flowering time regulation by flower bud differentiation is defective, and there has been no research on flowering initiation.

*Magnolia* × *soulangeana* ‘Changchun’ belongs to *Magnolia* (Magnoliaceae). It has stable genetic characteristics of flowering in spring and summer, and the floral transition and flower initiation are actually two independent phenological and developmental processes [18]. In this study, taking advantage of the difference in gene expression levels between dormant and expanded flower buds during the spring and summer flowering initiations of ‘Changchun’, we will unscramble the molecular basis of flowering regulation in spring and summer from the perspective of flowering initiation by the BGISEQ-500 sequencing platform. The research results will provide new data with an analysis of the biological characteristics of multiple flowering phenomena in trees.

## 2. Materials and Methods 

### 2.1. Plant Materials

The ‘Changchun’, approximately 9 years old, used in this study was grown outdoors at Nanjing Forestry University (32°5′ N, 118°49′ E, Nanjing, China). The north subtropical humid climate has four distinct seasons with an annual average temperature of 15.4 °C. The rainfall is abundant, with an average rainfall of 1106.5 mm and a relative humidity of 76%. The ‘Changchun’ was maintained according to ordinary culture practices without any diseases and insect pests. 

### 2.2. Observation of Flowering Initiation

A total of four annual growth phenological observations were performed from 2015 to 2018. Thirty flower buds that grow well—disease-free and pest-free—were selected and labeled from the middle and upper regions of the tree crown. Subsequently, the length, width, and growth state of the flower buds were measured and recorded every 3–7 days by conventional morphological detection. At the same time, the flower buds with the same growth condition as the marked flower buds were collected and photographed immediately, with three biological repetitions. 

### 2.3. Sample Collection

**Figure 1:**
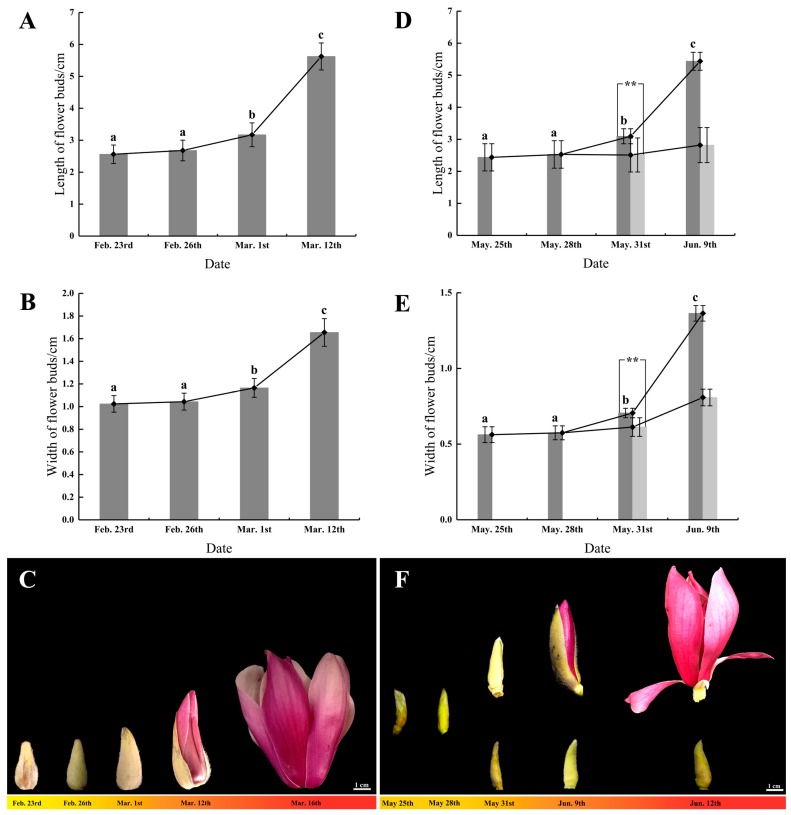
Morphological changes of flowering bud of ‘Changchun’. (**A**,**D**) The length growth of dormant, expanded, and red-tepal-exposed flower buds in spring flowering (**A**) and summer flowering (**D**); (**B**,**E**) the width growth of dormant, expanded, and red-tepal-exposed flower buds in spring (**B**) and summer (**E**); (**C**,**F**) the morphological changes of dormant, expanded, and red-tepal-exposed flower buds in spring (**C**) and summer (**F**). ** represent that the significant difference reached extremely significant level (*p* < 0.01).

According to the different stages of spring and summer flowering periods in ‘Changchun’, three types of flower buds were selected as samples: dormant flower buds, expanded flower buds, and red-tepal-exposed flower buds (Figure 1). Fresh flower buds of each type were collected randomly with three biological replicates (each biological replicate contained three flower buds), snap-frozen in liquid nitrogen, and stored at −80 °C until used for total RNA isolation.

### 2.4. RNA Extraction and cDNA Library Construction

According to Carolina et al., total RNA was extracted from collected flower bud samples using the improved CTAB-PVP method [26]. The concentration and quality of RNA was detected by a NanoDrop 8000 spectrophotometer and agarose gel electrophoresis, respectively. RNA purity and integrity were assessed using the RNA Nano 6000 Lab Chip Kit for the Agilent Bioanalyzer 2100 system (Agilent Technologies, CA, USA). The RNA integrity number (RIN) of >7.0 was used as the standard.

The poly-(A)-containing mRNA was enriched using magnetic beads with OligodT. The obtained RNA was disrupted by a fragmentation buffer. The random N6 primer was subjected to reverse transcription, and the second-strand cDNA was then synthesized to form double-stranded DNA. The double-stranded cDNA fragments were subjected to end repair and adapter ligation. The adapter-modified fragments were PCR amplified by specific primers, and a cDNA library was finally constructed.

We constructed 12 independent libraries, including ZFFU1, 2, 3; ZFFE1, 2, 3; ZSFU1, 2, 3; and ZSFE1, 2, 3, which were produced from each biological replicate of four types of flower buds (spring dormant flower buds (ZFFU), spring expanded flower buds (ZFFE), summer dormant flower buds (ZSFU), and summer expanded flower buds (ZSFE)).

### 2.5. Illumina Sequencing, De novo Assembly, and Annotation

The constructed cDNA libraries were sequenced on a BGISEQ-500 sequencing platform by the Beijing Genomics Institute (Shenzhen, China). The raw reads obtained from sequencing were counted by SOAPnuke and filtered using trimmomatic. After removing the adapter in raw reads, reads with ambiguous N nucleotides greater than 5%, and low-quality reads (bases with a mass value below 10 accounts for more than 20% of the total number of bases in the reads), the obtained filtered clean reads were saved in FASTQ (Fast Adaptive Shrinkage/Thresholding Algorithm with Qualities) format. The clean reads were used for de novo assembly by Trinity. The assembled transcripts were then clustered for removing redundancy using Tgicl, and unigenes were finally obtained. The assembled transcript was assessed for quality using a single copy ortholog database, BUSCO, and the integrity of the transcriptome assembly was explained to some extent by comparison with conserved genes.

For genes and proteins function annotations, all the assembled unigenes were queried against the KEGG (Kyoto Encyclopedia of Genes and Genomes), GO (Gene Ontology), NR (NCBI non-redundant protein sequences), NT (NCBI non-redundant nucleotide sequences), Pfam (Protein family), KOG (euKaryotic Orthologous Groups), SwissProt (A manually annotated and reviewed protein sequence database), TF (Transcription Factors), and PRG (Plant Resistance Gene) databases. 

### 2.6. Gene Quantification and Differential Gene Expression Analysis

The expression levels of genes were calculated using RSEM [27]. FPKM (the expected number of fragments per kilobase of transcript sequence per million base pairs sequenced) was used to quantify gene expression.

The differential gene expression between the two contrast groups was performed using the DESeq R software package. The DEGseq method was based on the Poisson distribution, and the differential genes were detected according to the method described by Wang et al. [28]. Genes with an adjusted *p*-value ≤ 0.001 and a fold change ≥ 2 or fold change ≤ 0.5 were defined as significantly DEGs using the Benjamini and Hochberg approach [29].

### 2.7. qRT-PCR Verification and Expression Analysis

According to Fan et al. [25], total RNA was extracted from dormant, expanded, and red-tepal-exposed flower buds using an RNAprep Pure Plant Kit (TaKaRa, Dalian, China). After treatment with DNase, the total RNA was subjected to reverse transcriptase reactions with the PrimeScript RT (Reverse Transcription) Reagent Kit (TaKaRa, Dalian, China) according to the manufacturer’s protocol. The primers were designed using Primer 5.0 (Table S1). The qRT-PCR experiments were performed using an SYBR Premix Ex Taq Kit (TaKaRa) on the ABI (Applied Biosystems) StepOne Plus Real Time System. *UBC* (Ubiquitin-conjugating enzyme gene) and *GBP* (gene encoding GTP-binding protein) were used as the reference genes (Table S2). The PCR program was as follows: 30 s at 95 °C, followed by 40 cycles of 5 s at 95 °C and 30 s at 60 °C The relative expression level of the target genes was calculated by the 2^−^^△△^^Ct^ method [30].

### 2.8. Data Statistics and Analysis

Sequencing data of three biological replicates from each type of flower buds were integrated, and renamed as ZFFU, ZFFE, ZSFU, and ZSFE, respectively, and then, used for further analysis.

Using Photoshop CS5 software (Adobe Systems Incorporated, CA, US) for plate making, and Origin 2018 performs analysis of Venn charts and heat maps. Both phenological morphology observation data and qRT-PCR experimental data were statistically analyzed and graphed using Excel 2010 (Microsoft Corporation, Washington, WA, US) and SPSS 21.0 (International Business Machines Corporation, Armonk, NY, USA).

## 3. Results

### 3.1. The Developmental Status of Flower Buds during Flowering Initiation

#### 3.1.1. Flower Bud Growth and Morphological Changes in Spring Flowering

From February 23 to February 26, the flower buds of ‘Changchun’ were oval and showed no significant change (Figure 1C). The length and width increased by 4.64% and 1.95%, respectively (Figure 1A,B). From February 26 to March 1, the shape of the flower buds was significantly enlarged (Figure 1C), and the length and width increased by 18.36% and 11.59%, respectively (Figure 1A,B). The flower bud growth showed an extremely significant difference (*p* < 0.01). It was assumed that this was the spring expanded flower bud stage. From March 1 to 12, due to the rapid growth of the flower buds, the external bracts were cracked, and the red tepals of flower buds were exposed, which meant that the red-tepal-exposed stage was imminent (Figure 1A–C). Finally, flower buds bloomed on March 16 (Figure 1C).

#### 3.1.2. Flower Bud Growth and Morphological Changes in Summer Flowering

From May 25 to 28, the flower buds of ‘Changchun’ showed a long cone shape with no obvious morphological changes (Figure 1F). The length and width only increased by 3.69% and 2.13%, respectively (Figure 1D,E), which suggested that the flower buds at this stage exhibited dormancy-like characteristics. From May 28 to 31, the flower buds showed two growth trends. Some flower buds showed no obvious changes in morphology and growth, and the other part of the flower buds expanded significantly because the length and width increased by 22.43% and 22.78%, respectively (Figure 1D–F), which indicated an extremely significant difference (*p* < 0.01). A *t*-test showed that over a given period, the growth amount of the expanded flower buds was significantly higher than that of the unexpanded flower buds (*p* < 0.01). It was presumed that the summer expanded flower bud stage began. From May 31 to June 9, due to the rapid growth of flower buds, the external bracts were cracked. The red tepals of the flower buds were exposed, which represented the red-tepal-exposed stage (Figure 1D–F). Finally, flower buds bloomed on June 12 (Figure 1F).

### 3.2. Transcriptome Sequencing and Function Annotation of Spring and Summer Flowering Initiation 

We sequenced RNA samples extracted from spring dormant flower buds (ZFFU), spring expanded flower buds (ZFFE), summer dormant flower buds (ZSFU), and summer expanded flower buds (ZSFE), resulting in 77.20, 74.71, 74.71, and 76.37 million raw reads in the four libraries, respectively. After removing low-quality sequences and ambiguous reads, approximately 71.00, 68.64, 67.69, and 69.09 million clean reads were obtained in the four libraries mentioned above, and the yields of clean reads were over 90%. The Q20 and Q30 in different libraries were greater than 97% and 89%, separately, indicating that the quality of the clean reads was high (Table 1, Table S10). Through the de novo assembly analysis of Trinity software, the number of unigenes obtained by ZFFU, ZFFE, ZSFU, and ZSFE libraries was 55,786; 53,792; 63,000 and 54,682, respectively. The average size of the unigenes was generally between 900 bp and 1100 bp, wherein about 80% of the unigene lengths in each library were distributed in the range of 200–2000 bp. The unigenes N50 length was between 1600 bp and 1700 bp, and the GC content was above 40% (Table 2, Table S11).

**Figure 2:**
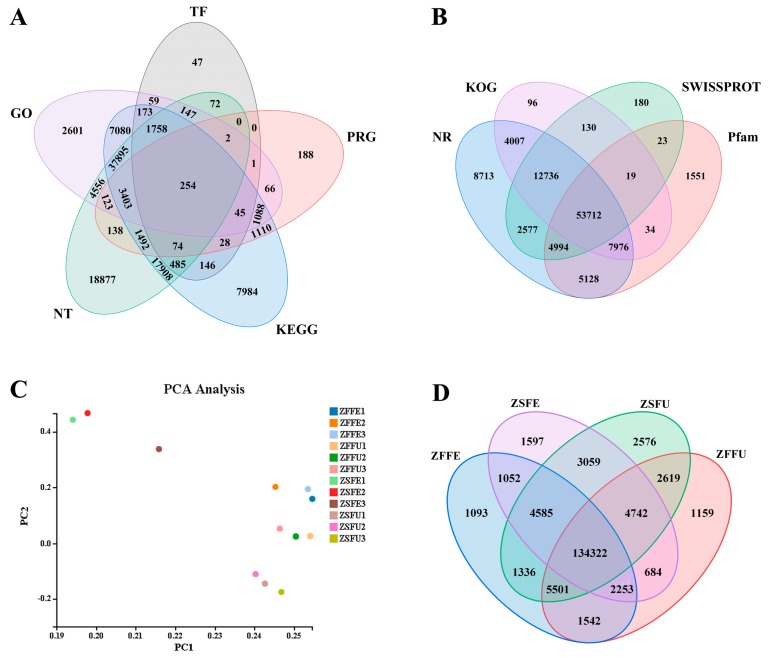
Functional annotation and expression analysis of genes. (**A**,**B**) Venn diagrams of expressed genes annotated in the nine gene and protein function databases; (**C**) The principal component analysis of expressed genes in different samples; (**D**) Venn diagram of gene number in four libraries. ZFFU = spring dormant flower buds; ZFFE = spring expanded flower buds; ZSFU = summer dormant flower buds; ZSFE = summer expanded flower buds; ZFFU1, ZFFU2, and ZFFU3 refer to three biological repetitions of ZFFU; ZFFE1, ZFFE2, and ZFFE3 refer to three biological repetitions of ZFFE; ZSFU1, ZSFU2, and ZSFU3 refer to three biological repetitions of ZSFU; ZSFE1, ZSFE2, and ZSFE3 refer to three biological repetitions of ZSFE.

Against nine major public function databases, 168,120 unigenes were compared to obtain annotations. The results showed that 59.39%, 51.86%, 44.24%, 48.13%, 46.82%, 43.68%, 35.24%, 1.96%, and 4.77% of unigenes showed significant similarities to known genes and proteins in the NR, NT, SwissProt, KEGG, KOG, Pfam, GO, TF, and PRG databases, respectively (Table 3). Venn analysis showed that all genes obtained at least one annotation in the nine databases. Annotations were obtained for 254 genes simultaneously for GO, NT, KEGG, TF, and PRG databases (Figure 2A), while 53,712 genes were annotated in SwissProt, Pfam, NR, and KOG databases simultaneously (Figure 2B).

### 3.3. Gene Expression Analysis of Spring and Summer Flowering Initiation

Principal component analysis was carried out on the gene expression data from spring and summer flowering initiation of ‘Changchun’. It can be seen that the biological repetitions in the three libraries, including ZFFU, ZFFE, and ZSFU, all showed good reproducibility, except for the ZSFE library, because ZSFE3 showed outliers (Figure 2C).

The number of genes expressed in ZFFU, ZFFE, ZSFU, and ZSFE was 152,822; 151,684; 158,740 and 152,294, respectively (Table S12). There were 1159, 1093, 2576, and 1597 genes (accounting for about 1%) specifically expressed separately in these four libraries (Figure 2D), which may be related to individual differences between dormant and expanded flower buds in spring and summer [18]. Nevertheless, there were 134,322 genes co-expressed in the four libraries, and the proportion of these genes in these four libraries was as high as 84.61–88.55%, indicating the apparently common characteristics of the gene expression of spring and summer flowering initiation. The reason for these results were not only related to tissue consistency, but also to the developmental state and pattern of the dormant and expanded flower buds in spring and summer, which also laid a good foundation for the recent screening of differential genes related to spring and summer flowering initiation.

### 3.4. Shared DEGs and Function Analysis of Spring and Summer Flowering Initiation

**Figure 3:**
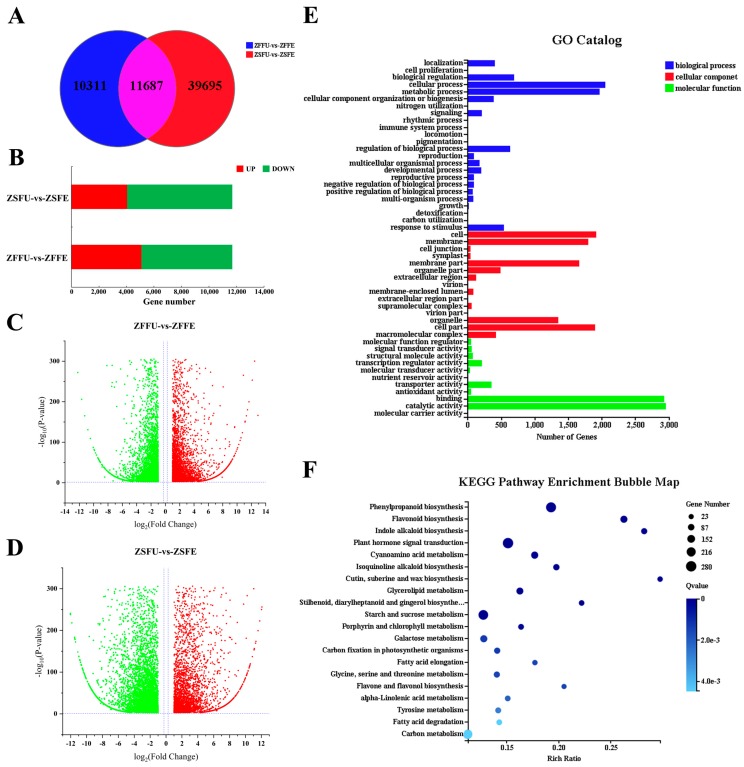
Number and function analysis of DEGs. (**A**) The Venn chart of DEGs in contrast groups of ZFFU vs. ZFFE and ZSFU vs. ZSFE; (**B**) up and down statistics of shared DEGs; (**C**,**D**) the volcanic map of shared DEGs during spring (**C**) and summer (**D**) flowering initiation; (**E**,**F**) the GO catalog (**E**) and the KEGG pathway enrichment bubble map (**F**) of shared DEGs of spring and summer flowering initiation. ZFFU vs. ZFFE: contrast group of spring flowering initiation; ZSFU vs. ZSFE: contrast group of summer flowering initiation.

In spring and summer flowering initiation, 21,998 and 51,382 significant DEGs (fold change ≥ 2 or fold change ≤ 0.5; adjusted *p*-value ≤ 0.001) were identified, respectively, and 11,687 were shared DEGs (Figure 3A, Table S3). In terms of expression pattern, 5093 and 4048 genes in the shared DEGs were significantly up-regulated, while 6594 and 7639 genes were significantly down-regulated in expanded flower buds of spring and summer, compared with dormant flower buds (Figure 3B, Table S3). The absolute value of log_2_ (fold change) of shared DEGs was between 1 and 5 during spring and summer flowering initiation, and that of a small number of genes could even reach between 10 and 14 (Figure 3C,D, Table S3). From the above results, we can see that the expression patterns of shared DEGs are relatively consistent both in spring and summer flowering initiation, and the differential fold change levels are obvious. Therefore, it is speculated that the shared DEGs may participate in the regulation of flowering initiation in spring and summer.

GO functional classification of shared DEGs showed that they could be divided into three major categories—biological processes, cell components, and molecular functions—and 50 minor classes. In biological processes, genes were enriched significantly in biological regulation (690), cellular process (2053), metabolic process (1964), and biological process regulation (635). In cell components, genes were involved in cell and cell parts (1916/1895), membranes and membrane parts (1793/1661), organelles (1351), and other functions. In molecular function, genes were associated with binding (2923) and catalytic activity (2949) (Figure 3E, Table S4). 

The results of the KEGG pathway enrichment showed that genes involved in plant hormone signaling were highest in number, amounting to 280, followed by phenylpropanol biosynthesis and starch and sucrose metabolism, amounting to 261 and 246, respectively. In terms of enrichment level, the indole alkaloid biosynthesis and the cutin, suberine, and wax biosynthesis pathways had the highest gene enrichment level. The enrichment significance level showed that the gene enrichment of most pathways could reach extremely significant enrichment levels (Q-value < 0.01) (Figure 3F, Table S5).

Flowering is controlled by both endogenous and exogenous signals [31]. Through the functional analyses of DEGs, we proposed that endogenous signals function as the main regulators in flowering initiation of ‘Changchun’. On the one hand, the phenylpropanol biosynthesis and the starch and sucrose metabolism might provide a certain material and energy basis for the flower buds expansion, and might relate to significant changes in the external morphology of flower buds when the flowering was initiated. On the other hand, the endogenous plant hormone signaling plays a critical role in triggering spring and summer flowering initiation of ‘Changchun’.

### 3.5. DEGs Related to Gibberellin Signaling in Spring and Summer Flowering Initiation

**Figure 4:**
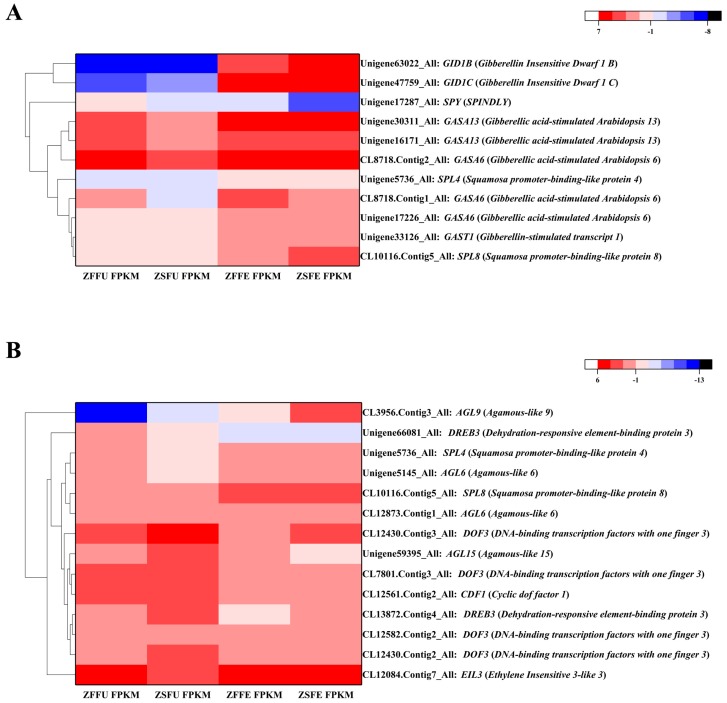
Heatmap of shared DEGs related to flowering. (**A**) The heat map of DEGs putatively associated with gibberellin signaling; (**B**) the heat map of DEGs putatively associated with transcription factors. ZFFU = spring dormant flower buds; ZFFE = spring expanded flower buds; ZSFU = summer dormant flower buds; ZSFE = summer expanded flower buds.

Among the shared DEGs, *GID1B* (*GIBBERELLIN INSENSITIVE DWARF1 B*) and *GID1C* encoding gibberellin (GA) receptors were significantly up-regulated both in expanded flower buds of spring and summer. The log_2_ (fold change) of *GID1B* was 12.17 and 13.67, and the log_2_ (fold change) of *GID1C* was 11.27 and 9.99, respectively. *SPY* (*SPINDLY*), a negative regulatory gene of a GA signaling, showed significant down-regulation in spring and summer expanded flower buds, and the log_2_ (fold change) of *SPY* was −1.31 and −4.18, respectively. Parts of the genes encoded GASA (GIBBERELLIC ACID-STIMULATED ARABIDOPSIS) and GAST (GA-STIMULATED TRANSCRIPT) proteins, whose family members were regulated by GA. The expression levels of *GAST1*, *GASA6,* and *GASA13* increased in expanded flower buds of spring and summer, and their log_2_ (fold change) values were all greater than 1. As a response gene of the GA pathway signaling, the *SPL* gene family had two members, *SPL4* and *SPL8*, which showed significantly higher expression during spring and summer flowering initiation. The former log_2_ (fold change) was 1.06 and 1.30, and the latter was 1.00 and 2.37, respectively (Figure 4A, Table S6). Therefore, it may be speculated that the spring and summer flowering initiation of ‘Changchun’ is related to gibberellin signaling.

### 3.6. Related Transcription Factors in Spring and Summer Flowering Initiation

The results of sequencing analysis indicated that AGL (AGAMOUS-LIKE) members were dominant in the MADS-box transcription factor family, of which *AGL6* and *AGL9* were up-regulated both in expanded flower buds of spring and summer, while *AGL15* was down-regulated. *CDF1* (*CYCLIC DOF FACTOR 1*) and *DOF3*, belonging to the DOF (Dof zinc finger protein) family, displayed significant down-regulation both in expanded flower buds of spring and summer. The EIL (ETHYLENE INSENSITIVE 3-like) transcription factor family might be involved in the ethylene signaling transduction, and its member, *EIL3* (*ETHYLENE INSENSITIVE* 3-like *3*), exhibited significantly higher expression during spring and summer flowering initiation. Two members of the SBP (SQUAMOSA PROMOTER BINDING PROTEIN) family that were involved in flowering regulation, *SPL4* and *SPL8*, were up-regulated in expanded flower buds. The number of AP2-EREBP family members was the highest, and the expression of *DREB3* (*DEHYDRATION-RESPONSIVE ELEMENT-BINDING PROTEIN 3*), which might be involved in flowering regulation, revealed down-regulation in expanded flower buds (Figure 4B, Table S7).

### 3.7. Expression Verification of Genes Related to Spring and Summer Flowering Initiation

**Figure 5:**
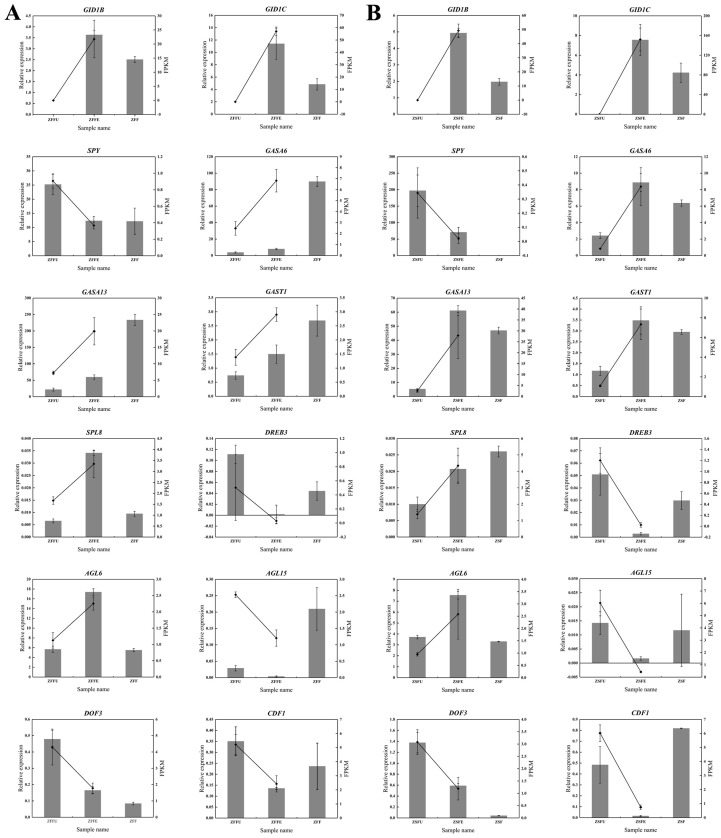
qRT-PCR analysis of 12 DEGs. (**A**) The line charts and histograms of qRT-PCR and transcriptome expression of 12 DEGs in spring flowering initiation; (**B**) the line charts and histograms of qRT-PCR and transcriptome expression of 12 DEGs in summer flowering initiation. Using qRT-PCR technology, we examined the relative expression levels of 12 DEGs in spring and summer dormant and expanded flower buds to verify the accuracy of transcriptome sequencing. Meanwhile, we also examined the relative expression levels of these genes in spring and summer red-tepal-exposed flower buds to exhibit their expression changes during spring and summer flowering. The line charts and histograms represent the expression level of 12 DEGs in transcriptome sequencing and qRT-PCR analysis, respectively. The left y-axis represents the relative expression from the qRT-PCR results, while the right y-axis represents the FPKM value from the RNA-Seq. ZFFU = spring dormant flower buds; ZFFE = spring expanded flower buds; ZFF = flower buds that red tepals were exposed in spring; ZSFU = summer dormant flower buds; ZSFE = summer expanded flower buds; ZSF = flower buds that red tepals were exposed in summer.

The expression patterns of 12 genes putatively involved in gibberellin signaling and flowering-initiation-related transcription factors in dormant, expanded, and red-tepal-exposed flower buds of spring and summer were verified by real-time quantitative PCR. The results showed that the expression patterns of these genes during spring and summer flowering initiation were basically consistent with those obtained by RNA-seq, indicating that the sequencing results were reliable (Figure 5). Among the 12 genes, the expression levels of *GID1B* and *GID1C* were both the highest in the expanded flower buds, indicating that these two might be specific regulators of spring and summer flowering initiation. *SPY* expression levels continued to decline and remained low, which might be related to the activation of gibberellin signaling. The expression level of *SPL8* was highest in spring expanded flower buds and in summer red-tepal-exposed flower buds. *GASA6, GASA13,* and *GAST1* exhibited constant up-regulation during spring flowering, while their expression levels in summer were only highest in the expanded flower buds, indicating that these genes might play a specific regulatory role in summer flowering initiation. Among the flowering initiation putative transcription factors, *AGL15*, *DREB3*, and *CDF1* all showed the lowest expression levels both in the expanded flower buds of spring and summer, while the expression of *AGL6* was the highest in the expanded flower buds. These results indicate that they might specifically participate in the regulation of spring and summer flowering initiation (Figure 5).

## 4. Discussion

### 4.1. Flowering Initiation Is an Important Stage during ‘Changchun’ Flowering

Flowering is a crucial event during reproductive growth in woody plants, which is essential for plant reproduction and hereditary trait transmission [32]. The characteristic of flowering many times can highlight the ornamental effects of woody plants. Flower bud differentiation was generally considered to be a symbol of flowering in recent studies on plant flowering regulation, such as *Arabidopsis thaliana*, *Camellia azalea* [33], and *Carya cathayensis* [6]. During the annual growth and development of ‘Changchun’, some flower buds formed by reproductive transformation begin to flower in summer, while the remaining flower buds grow slowly. After overwintering, the flower buds will break up dormancy, and then, start to flower in the following spring [18]. This specific flowering pattern differs from that in which ‘Old Blush‘ rose can be converted multiple times between vegetative and reproductive growth in a year [16].

Previous research has suggested that the openness of flower buds in woody plants was related to dormancy release [13,14,15]. Before the spring flowering of ‘Changchun’, the long dormancy process of flower buds was released, and the growth amount increased significantly. The external morphology then changed. This process is similar to that of *Pyrus pyrifolia* [14,34], *Prunus persica* [35], and *Prunus armeniaca* [36]. It is worth noting that part of the flower buds of ‘Changchun’ do not undergo a long dormancy process after reproductive transformation in summer, but only show a short dormancy-like process and then rapidly expand and flower. The different characteristics of flower bud development after floral transition indicate that the woody plants can flower without undergoing dormancy. It has also been shown that the floral transition and flowering initiation of woody plants are two distinctly independent stages.

### 4.2. Gibberellin Signaling Participates in Spring and Summer Flowering Initiation

Gibberellin plays an important role in plant growth and development, especially in floral transition and flowering time regulation [37]. GID1 is a receptor for gibberellin. There are three types of GA receptors in *Arabidopsis thaliana*, GID1A–C, which are functionally redundant and can sense and combine with GA. The GID1–GA complex interacts with DELLA, resulting in ubiquitination and degradation, thereby producing a gibberellin effect [38,39]. In the spring and summer flowering initiation of ‘Changchun’, *GID1B* and *GID1C* were both hardly detected in the dormant flower buds, but their expression sharply increased in the expanded flower buds and retained relatively high levels until the red-tepal-exposed stage. DELLA proteins are negative regulators of GA signaling [40]. There are four DELLA protein involved in the negative regulation of flowering in *Arabidopsis thaliana*, including *GAI* (*GIBBERELLIC ACID INSENSITIVE*), *RGA* (*REPRESSOR OF ga1-3*), *RGL1* (*RGA-LIKE 1*) and *RGL2* [41]. We found that *RGL1-LIKE* showed significantly down-regulated during summer flowering initiation. Another DELLA protein encoding gene, *GAI*, also exhibited lower expression levels in expanded flower buds during spring flowering initiation, while its log_2_ (fold change) was –0.5 (*p*-value < 0.001, Q-value < 0.001) (Table S8, Table S9). SPY, which is upstream of DELLA, is another important player in the GA signal and exists as a negative regulator. *Spy* mutants exhibit an early flowering phenotype [42,43]. We found that the expression level of *SPY* was significantly down-regulated during the spring and summer flowering initiation, and remained at a low level until the red-tepal-exposed stage. These genes were mainly involved in the upstream of gibberellin signaling and might be related to the activation of GA signal transduction. 

Gibberellin signaling can exert its temporal and spatial regulation of flowering through downstream response genes [37]. *SPL* is a target gene downstream of DELLA. The accumulation of DELLA may result in the transcriptional repression of *SPL3*, *SPL4*, and *SPL5*. In turn, the reduced *SPL* activity causes a reduction in the expression of gibberellin signal integration genes and ultimately delays flowering [44]. Moreover, SPL8 is involved in reproductive development because of its positive role in GA-mediated anther development [45]. During the spring and summer flowering initiation of ‘Changchun’, *SPL4* and *SPL8* were both significantly up-regulated, and the expression level of *SPL8* was relatively high at the expanded flower bud stage and red-tepal-exposed stage, respectively. In addition, most members of the GASA family are regulated by gibberellin, which have important regulatory effects on flower development and flowering. Some *GASA* genes are also identified as target genes downstream of gibberellin signaling pathways [46,47,48]. However, there are a few studies on their involvement in flowering regulation in response to gibberellin signaling. According to the available research, *GASA6* can be up-regulated in response to gibberellin, and overexpression of it initiates early flowering in *Arabidopsis*. *GASA6*, as a target gene downstream of DELLA, may participate in gibberellin signaling and regulate flowering [48,49]. Tomato *GAST1* is the first identified as a gibberellin-induced gene [50]. The *GASA13* gene is also up-regulated by gibberellin in *Arabidopsis thaliana* [47,48]. In the process of spring and summer flowering initiation, the up-regulated expression patterns of *GASA6*, *GAST1,* and *GASA13* were highly consistent with the above studies. However, the highest expression levels of these three genes appeared, respectively, at the red-tepal-exposed stage in spring and expanded stage in summer. Whether or not these three genes participate in the response of gibberellin signaling in different ways requires further research.

### 4.3. Transcription Factors Participate in Spring and Summer Flowering Initiation 

Some transcription factors found in this study may be involved in flowering initiation regulation. They were mainly distributed in five transcription factor families, including AP2-EREBP, DOF, EIL, MADS, and SBP, which may regulate flowering by responding to hormones or environmental signals. Available research data show that *DREB3* genes can encode AP2/EREBP-type transcription factors, and their constitutive overexpression may improve frost tolerance of transgenic plants, but leads to the delay of flowering compared to control plants [51,52]. *DREB3* was down-regulated significantly in the spring and summer flowering initiation of ‘Changchun’. DOF transcription factors are involved in many plant-specific regulatory processes, and several *Arabidopsis* DOF transcription factors, such as CDF1/2/3, are associated with circadian rhythms and photoperiods, and their expression at high levels is sufficient to inhibit flowering. Furthermore, JcDof3 may also be involved in flowering time regulation, and may act as a negative regulator in the photoperiodic flowering pathway in *Jatropha curcas* [53,54,55]. For ‘Changchun’, the circadian rhythm of spring and summer flowering is obviously different. However, both *DOF3* and *CDF1* are significantly down-regulated. Therefore, whether these transcription factors participate in the regulation of flowering initiation in spring and summer flowering initiation in a relatively specific way still needs further study. The regulation of ethylene on flowering varies from species to species [56,57]. The transcriptional response to ethylene includes the EIL families of transcription factors (TFs) [58]. The up-regulated expression of *EIL3* in expanded flower buds suggests that it may positively regulate the spring and summer flowering initiation. Some members of the MADS-box transcription factor family, such as AGL6, AGL9 [59,60], and AGL15, play important roles in flower development and flowering time regulation. AGL6 is relevant to the identification of floral organs [61]. The expression of *AGL6*-LIKE can be increased by gibberellin induction, and transgenic plants that ectopically expressed the *AGL6*-LIKE gene can flower earlier than wild-type plants [62,63]. It is speculated that *AGL6* may be involved in the amplification of gibberellin signaling to regulate the initiation of flowering in spring and summer. AGL15 has been shown to act as a floral repressor in *Arabidopsis thaliana* [64]. Double mutants of *AGL15* and *AGL18* flower early under non-inductive conditions [65]. This indicates that the down-regulated expression of *AGL15* specifically regulates the spring and summer flowering initiation of ‘Changchun’. The up-regulation of *SPL4* and *SPL8* expression further suggests that certain members of the SBP family may regulate flowering time by responding to gibberellin signaling.

## 5. Conclusions

The flowering initiation of woody plants is an important stage in the flowering process, and is an independent period of the flower bud differentiation. This study showed that during spring and summer flowering initiation, the growth of expanded flower buds increased significantly, and the shape of which was also significantly enlarged, indicating that flowering was initiated. In accordance with this phenomenon, we constructed transcriptome libraries of spring and summer flowering initiation in ‘Changchun’, and screened the shared DEGs of spring and summer flowering initiation. Many genes were mainly involved in plant hormone signaling. The differential expression patterns of *GID1B*, *GID1C*, *SPL8*, *SPL4*, *SPY*, *GASA6*, *GASA13*, and *GAST1* indicated that the regulation of spring and summer flowering initiation in ‘Changchun’ was closely related to gibberellin signaling. Furthermore, AP2-EREBP, DOF, EIL, MADS, and SBP family-associated transcription factors were also involved in the regulation of flowering initiation in spring and summer. The results of qRT-PCR analysis confirmed the transcriptional level changes of key regulatory genes involved in spring and summer flowering initiation, indicating that the BGISEQ-500 sequencing results were true and reliable, and speculated that *GID1B*, *GID1C,* and *AGL6*, *AGL15*, *DREB3*, and *CDF1* may specifically participate in the regulation of spring and summer flowering initiation. 

Obviously, spring and summer flowering initiation of ‘Changchun’ was involved in the specific regulation of gibberellin-related genes and transcription factors. This study represents a first step towards illuminating the molecular mechanisms of spring and summer flowering initiation in ‘Changchun’, and the results indicate a new perspective for the study of flowering initiation regulation in perennial woody plants.

## Figures and Tables

**Table 1:** Overview of reads filtering and quality in four libraries.

Sample	Raw Reads (M)	Clean Reads (M)	Q20 (%)	Q30 (%)
ZFFU	77.20	71.00	97.62	89.75
ZFFE	74.71	68.64	97.57	89.36
ZSFU	74.71	67.70	97.98	90.46
ZSFE	76.37	69.09	97.84	89.99

**Table 2:** Overview of unigenes quality in four libraries.

Sample	Total Number	Mean Length	200–2000 nt	N50	GC (%)
ZFFU	55786.33	1094.33	84.90%	1690.00	44.13
ZFFE	53792.00	1087.67	85.18%	1683.00	44.19
ZSFU	63000.67	986.33	87.28%	1627.67	43.89
ZSFE	54682.00	1045.67	85.86%	1692.33	44.17

**Table 3:** Overview of unigenes function annotation.

Values	Total	NR ^1^	NT ^2^	Swissprot ^3^	KEGG ^4^
Number	168120	99843	87184	74371	80923
Percentage	100%	59.39%	51.86%	44.24%	48.13%
Values	KOG ^5^	Pfam ^6^	GO ^7^	TF ^8^	PRG ^9^
Number	78710	73437	59251	3291	8012
Percentage	46.82%	43.68%	35.24%	1.96%	4.77%

^1^ NCBI non-redundant protein sequences; ^2^ NCBI non-redundant nucleotide sequences; ^3^ A manually annotated and reviewed protein sequence database; ^4^ Kyoto Encyclopedia of Genes and Genomes; ^5^ clusters of euKaryotic Orthologous Groups; ^6^ Protein family; ^7^ Gene Ontology; ^8^ Transcription Factors; ^9^ Plant Resistance Gene.

## Supplementary Materials

The following are available online at https://www.mdpi.com/2073-4425/11/1/15/s1, Table S1: Primers used to analyze the expression of the gibberellin signaling and transcription factor related genes and the reference genes. Table S2: Detection of internal reference gene expression stability by geNorm, NormFinder, and BestKeeper software. Table S3: All the shared differentially expressed genes in four libraries. Table S4: GO catalog of the shared differentially expressed genes. Table S5: KEGG pathway enrichment of the shared differentially expressed genes. Table S6: Expression patterns, levels, and function annotation of gibberellin signaling related genes in spring and summer flowering initiation. Table S7: Expression patterns, levels, and function annotation of transcription factor related genes in spring and summer flowering initiation. Table S8: Expression level and annotations of *GAI*. Table S9: Expression level and annotations of *RGL1*-like. Table S10: Sequencing data quality statistics results; Table S11: Unigene quality statistics results. Table S12: Gene expression statistics in 12 libraries.
